# A simple non-invasive method to collect soft tick saliva reveals differences in *Ornithodoros moubata* saliva composition between ticks infected and uninfected with *Borrelia duttonii* spirochetes

**DOI:** 10.3389/fcimb.2023.1112952

**Published:** 2023-01-20

**Authors:** Serhii Filatov, Filip Dyčka, Jan Sterba, Ryan O.M. Rego

**Affiliations:** ^1^ National Scientific Center "Institute of Experimental and Clinical Veterinary Medicine", Kharkiv, Ukraine; ^2^ Institute of Parasitology, Biology Centre of the Czech Academy of Sciences, Ceske Budejovice, Czechia; ^3^ Faculty of Science, University of South Bohemia, Ceske Budejovice, Czechia

**Keywords:** tick, *Borrelia duttonii*, *Ornithodoros moubata*, saliva, LC-MS analysis, infected, artificial membrane feeding, relapsing fever spirochetes

## Abstract

**Introduction:** We developed a new simple method to assess the composition of proteinaceous components in the saliva of *Ornithodoros moubata*, the main vehicle for pathogen transmission and a likely source of bioactive molecules acting at the tick-vertebrate host interface. To collect naturally expectorated saliva from the ticks we employed an artificial membrane feeding technique using a simple, chemically defined diet containing phagostimulants and submitted native saliva samples collected in this way for liquid chromatography-mass spectrometry (LC-MS) analysis. These experiments were conducted with groups of uninfected ticks as well as with *O. moubata* infected with *B. duttonii*. The ticks exhibited a fair feeding response to the tested diet with engorgement rates reaching as high as 60-100% of ticks per feeding chamber. The LC-MS analysis identified a total of 17 and 15 proteins in saliva samples from the uninfected and infected *O. moubata* nymphs, respectively. Importantly, the analysis was sensitive enough to detect up to 9 different proteins in the samples of saliva containing diet upon which as few as 6 nymphal ticks fed during the experiments. Some of the proteins recognized in the analysis are well known for their immunomodulatory activity in a vertebrate host, whereas others are primarily thought of as structural or “housekeeping” proteins and their finding in the naturally expectorated tick saliva confirms that they can be secreted and might serve some functions at the tick-host interface. Most notably, some of the proteins that have long been suspected for their importance in the vector-pathogen interactions of *Borrelia* spirochetes were detected only in the samples from infected ticks, suggesting that their expression was altered by the persistent colonization of the tick’s salivary glands by spirochetes. The simple method described herein is an important addition to the toolbox available to study the vector-host-pathogen interactions in the rapidly feeding soft ticks.

## Introduction

The salivary route is one of the major modes of transmission for tick-borne pathogens (Schwan and Piesman, 2002). Thus, the feeding physiology of ticks is a very important but rather understudied topic. For example, many different bioactive compounds such as lipids and proteins have been identified in tick salivary glands and chemically induced salivary secretions but their exact roles in the vector-host-pathogen interactions remain largely unknown (Oliveira et al., 2011; Mans, 2019). Soft ticks of the *Ornithodoros moubata* species complex are primarily known as vectors of African swine fever virus (ASFV) and Relapsing fever (RF) spirochete, *Borrelia duttonii* across their distributional range in Africa (Bakkes et al., 2018). Both pathogens when present within a territory severely affect livelihoods, especially in poor agricultural communities, and have an epidemic potential to spread far beyond originally affected countries (Cutler, 2006; Chenais et al., 2019; Blome et al., 2020). This justifies efforts to unravel the complex molecular interactions at the vector-host-pathogen interface that could allow researchers to come up with innovative interventions such as anti-tick or transmission-blocking vaccines to prevent the perpetuation of these diseases.

Owing to their epidemiological importance and thanks to the widespread availability of *O. moubata* sensu lato ticks in laboratory colonies across the world, an impressive amount of work has been carried out on this species group to characterize the composition of saliva and identify biochemical properties for some of its constituents (Baranda et al., 1997; Díaz-Martín et al., 2013; Bernard et al., 2016; Mans et al., 2019; Oleaga et al., 2021a; Oleaga et al., 2021b). However, most of these studies used either dissected salivary gland extracts or chemically induced salivary secretions from multiple adult ticks to increase the yield of proteins, which is not a very realistic scenario of what occurs *in vivo*, during the transmission event. Moreover, for a long time, it has been suspected that, at least in the case of *B. duttonii*, different life stages of *O. moubata* transmit the pathogen through distinct routes, with adult ticks transmitting spirochetes predominantly *via* contamination of feeding lesions by infected coxal fluid, and nymphal stages transmitting through the salivary route (Schwan and Piesman, 2002).

It is not known whether the salivary gland environment or saliva composition is substantially different between the life stages, but since instars are the most appropriate stages to begin with characterizing factors underlying the salivary transmission of *B. duttonii* we hypothesized that: (i) using an artificial feeding system and a simple, chemically defined diet containing phagostimulants it will be possible to collect naturally expectorated saliva from *O. moubata* nymphs; (ii) it will be possible to identify and compare proteins secreted by *B. duttonii* infected vs uninfected nymphs. The results of our pilot study and a brief discussion regarding the possible biological significance of the observed differences are presented below.

## Methods

### Tick maintenance and generation of infected ticks

Ticks used in the experiments were *O. moubata* nymphal stages 2-4 (N2-4) maintained in the laboratory colony at the Institute of Parasitology, Biology Centre of the Czech Academy of Sciences (BC CAS), Ceske Budejovice, Czech Republic and were starved for at least 2 months before the experiments. The ticks are maintained at a temperature of 28°C and a Relative Humidity between 80-85% with a 14/10 light/dark period.

Infected ticks were generated in the course of a different study by feeding naive N1 stage ticks on C3H mice, intraperitoneally infected with the 1120K3 strain of *B. duttonii* (generously provided by Sven Bergstrom, Sweden), at peak spirochaetemia, a procedure which in our experience results in 100% infection rates of engorged ticks, as confirmed by injections of homogenates prepared from molted individual ticks into susceptible animals (R.O.M. Rego, unpublished results). Ticks infected in this manner were kept under standard conditions in the laboratory colony (T= 25 ± 3°C; RH=80-85%; 14:10 light: dark cycle) before being used in the experiments.

### Tick feeding

To artificially feed the ticks we used chambers described by Kröber and Guerin (2007) but with a piece of stretched Parafilm-M^®^ instead of silicone to form a membrane at the feeding unit’s bottom. To stimulate tick feeding we used a 0.01M solution of reduced glutathione (GSH) in 0.15M NaCl containing 1 mg/ml glucose with the pH adjusted to ≈ 7 by adding a few drops of 10% NaOH (Galun and Kindler, 1965; Galun and Kindler, 1968; Hokama et al., 1987). The solution was prepared under aseptic conditions using ultrapure water (Milli-Q^®^) and 2 ml of it was pipetted to cover a bottom of a well inside a sterile 6-Well Plate; each well accommodated a single feeding unit (FU). The plate was rested on support inside a water bath warmed to 38° C. We aimed at feeding at least 10-15 ticks per FU but because preliminary trials have shown that the feeding membrane was prone to breakage when exposed to this number of *O. moubata*, we decided to feed them in smaller groups sequentially. Ticks were placed inside in groups of 2-3 individuals/unit and allowed to feed to repletion for 1-1.5 hours, after which time they were retrieved and a new group was induced to feed on the same membrane. In case the integrity of the Parafilm was compromised, the membrane was carefully replaced with an extra effort being made to prevent spillage of any liquid present inside the chamber (i.e. excreted coxal fluid) from getting into the well with the GSH solution. After several feeding sessions were complete, the leftovers of the solution containing tick saliva were collected into sterile 1.5 ml tubes and stored at +4°C before they were subjected to the protein purification and proteomics analysis. This approach was taken to increase the ratio between the feeding solution and saliva deposited by individual ticks.

### Protein purification and in-solution digestion

Tick saliva samples were incubated in five fold volume excess of cold acetone supplemented with 0.07% β-mercaptoethanol at -20°C overnight. Next day, supernatant was removed by centrifugation (10 000 g, 20 min, 4°C). Precipitated proteins were dissolved in 20 µl of 100 mM ammonium bicarbonate containing 4 M urea by shaking at room temperature for 20 min. Proteins were then diluted by adding 140 µl of 100 mM ammonium bicarbonate and the solution was shaked for 40 min. Protein concentration was measured using a BCA Protein Assay Kit (Thermo Fisher Scientific, MA, USA).

Proteins were reduced with 10 mM dithiothreitol at 56°C for 40 min and alkylated with 55 mM iodoacetamide at room temperature in the dark for 20 min. The reaction was quenched by 55 mM dithiothreitol. The protein mixture was diluted to a final volume of 100 µl by adding 100 mM ammonium bicarbonate. Then samples were digested with trypsin at the ratio 50:1 at 37°C overnight. The digestion was terminated by the addition of formic acid to a final concentration of 5%. The obtained peptide mixtures were purified using C18 Empore™ disks (3M, MN, USA) (Rappsilber et al., 2007).

### Nano-LC-ESI-MS/MS

Peptides were dissolved in 30 µl of 3% acetonitrile/0.1% formic acid. The analysis was carried out on an UltiMate 3000 RLSCnano system (Thermo Fisher Scientific, MA, USA) coupled on-line to mass spectrometer timsTOF Pro (Bruker Daltonics, Bremen, Germany). The peptides were injected onto an Acclaim™ PepMap™ 100 C18 trapping column (300 µm i.d., 5 mm length, particle size 5 µm, pore size 100 Å; Thermo Fisher Scientific) using a 2 µl injection volume and a 2.5 µl/min flow rate for 2 min. The peptides were eluted from trapping column onto an Acclaim™ PepMap™ 100 C18 trapping column (75 µm i.d., 150 mm length, particle size 2 µm, pore size 100 Å; Thermo Fisher Scientific) and separated by a 48 min long linear gradient of 5-35% ACN/0.1% formic acid at a constant rate of 0.3 µl/min. Column oven temperature was set at 35°C. The MS analysis was operated in PASEF scan mode with positive polarity. Electrospray ionization was performed using a CaptiveSpray (Bruker Daltonics) with capillary voltage at 1500 V, dry gas at 3 l/min and dry temperature at 180°C. Ions were accumulated for 100 ms and 10 PASEF MS/MS scans were acquired per topN acquisition cycle. An ion mobility range (1/K0) was set at 0.6-1.6 Vs/cm^2^. Mass spectra were collected over a *m/z* range of 100 to 1700. A polygon filtering was applied to exclude the low *m/z* of singly charged ions. A target intensity was set at 20 000 to repeatedly select precursor for PASEF MS/MS repetitions. The precursors that reached the target intensity were than excluded for 0.4 min. Collision energies were changed from 20 to 59 eV in 5 steps of equal width between 0.6 and 1.6 Vs/cm^2^ of 1/K0 values.

### Proteomics data analysis

Raw MS data were processed by MaxQuant software (version 1.6.14) (Cox and Mann, 2008; Cox et al., 2011) with integrated Andromeda search engine (Cox et al., 2011). Database of *Ornithodoros moubata* downloaded from Uniprot (28. 10. 2020) and contaminant database included in MaxQant software were used to identify proteins. Default parameters for TIMS-DDA search type and Bruker TIMS instrument were applied. Trypsin/P was set as enzyme allowing up to two missed cleavages in specific digestion mode; carbamidomethylation of cysteine was used as fixed modification; methionine oxidation and protein N-term acetylation were set as variable modifications; the minimum required peptide length was set to seven amino acids. Precursor ion tolerance was set at 20 and 10 ppm in first and main peptide search, respectively; the mass tolerance for MS/MS fragment ions was set at 40 ppm; peptide spectrum match (PSM) and protein identifications were filtered using a target-decoy approach at a false discovery rate (FDR) of 1%. Label-free quantification (LFQ) of proteins was done using the algorithm integrated into MaxQuant with minimum ratio count set at 2.

Protein data tables obtained from MaxQuant were analysed using Perseus software (version 1.6.14.0) (Tyanova and Cox, 2018). Protein hits to the reverse database, contaminants and protein only identified with modified peptides were excluded from further analysis. Values of LFQ intensity were transformed by log base 2. Proteins with the number of identified razor peptides less than 2 along with the score lower or equal to 40 were filtered out of the data. The full dataset is available *via* ProteomeXchange with identifier PXD038824.

## Results

Both uninfected and *B. duttonii* infected ticks, when placed inside feeding chambers, readily ingested the GSH solution with engorgement rates reaching as high as 100% in some pools of ticks (**Table 1**). In total, 27 out of 45 (60%) versus 25 out of 28 (89%) individual ticks fed in uninfected and infected groups, respectively.

**Table 1:** Feeding response of *O. moubata* nymphs to GSH solution.

Feedings	Number of ticks fed (exposed)				
	FU1*	FU2*	FU3*	iFU1**	iFU2**
pool-1	1(3)	1(3)	0(3)	3(3)	3(3)
pool-2	2(3)	3(3)	0(3)	2(2)	2(2)
pool-3	1(3)	2(3)	3(3)	2(2)	3(3)
pool-4	3(3)	3(3)	1(3)	2(2)	0(2)
pool-5	3(3)	2(3)	2(3)	2(2)	3(3)
pool-6	–	–	–	1(2)	2(2)
Total	10(15)	11(15)	6(15)	12(13)	13(15)

* uninfected nymphs.

** nymphs infected with *B. duttonii* 1120K3.

**Figure 1:**
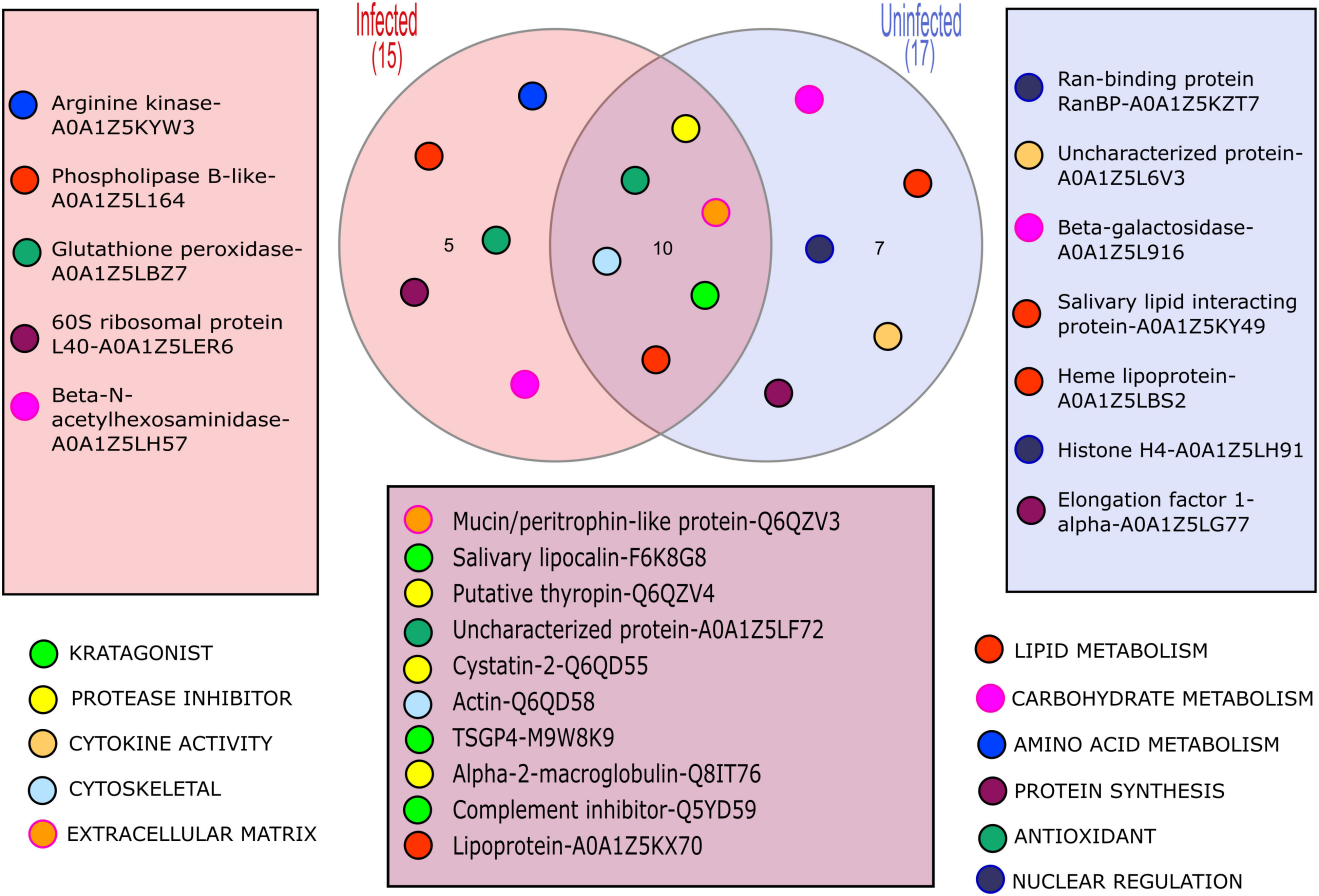
A Venn diagram showing the different proteins detected in samples from *B*. *duttonii* infected vs uninfected ticks, and their hypothetical functions.

The LC-MS/MS analysis was able to identify some proteins in the solution collected from each feeding unit; however, the number and relative amounts of detected proteins were substantially different between the FUs (**Supplemetary Figure 1**). Importantly, up to 9 proteins were identified in the samples of saliva containing solution upon which as few as 6 individual ticks fed during the experiments, which confirms the excellent sensitivity of the method. In total, we identified 22 proteins (**Figure 1**; **Table 2**) in the saliva containing samples collected from chambers used to artificially feed *O. moubata* nymphs.

**Table 2:** Proteins identified in the tick saliva samples.

#	UniProt accesion#	Protein name	Infected	Uninfected	Function (GO)	Putative role
1	A0A1Z5KX70	Lipoprotein (Fragment) OS=Ornithodoros moubata OX=6938 PE=4 SV=1	+	+	Lipid transporter activity	Housekeeping Metabolism/lipids
2	A0A1Z5KYW3 A0A1Z5LC39	**Arginine kinase (Fragment) OS=Ornithodoros moubata OX=6938 PE=3 SV=1;Arginine kinase (Fragment) OS=Ornithodoros moubata OX=6938 PE=3 SV=1**	+	–	Arginine kinase activity	Housekeeping Metabolism/energy
3	A0A1Z5L164	**Phospholipase B-like (Fragment) OS=Ornithodoros moubata OX=6938 PE=3 SV=1**	+	–	Phospholipase activity	Housekeeping Metabolism/lipids
4	A0A1Z5LBZ7	**Glutathione peroxidase (Fragment) OS=Ornithodoros moubata OX=6938 PE=3 SV=1**	+	–	Glutathione peroxidase activity	Housekeeping Detox/antioxidant
5	A0A1Z5LER6; A0A1Z5LGF2	**60S ribosomal protein L40 (Fragment) OS=Ornithodoros moubata OX=6938 PE=3 SV=1;40S ribosomal protein S27a (Fragment) OS=Ornithodoros moubata OX=6938 PE=3 SV=1**	+	–	Structural constituent of ribosome/Metal ion binding	Housekeeping Protein synthesis
6	A0A1Z5LF72	Uncharacterized protein (Fragment) OS=Ornithodoros moubata OX=6938 PE=3 SV=1	+	+	Oxidoreductase activity	Housekeeping Detox/antioxidant
7	Q6QD58; Q3LGW2; A0A1Z5LGC3; A0A1Z5LAN8	Actin OS=Ornithodoros moubata OX=6938 PE=2 SV=1;Actin OS=Ornithodoros moubata OX=6938 PE=2 SV=1;Actin Actin 1 (Fragment) OS=Ornithodoros moubata OX=6938 PE=3	+	+	–	Housekeeping Cytoskeletal
8	A0A1Z5LH57	**Beta-N-acetylhexosaminidase (Fragment) OS=Ornithodoros moubata OX=6938 PE=3 SV=1**	+	–	Beta-N-acetylhexosaminidase activity	Housekeeping Metabolism/carbohydrates
9	F6K8G8	Salivary lipocalin OS=Ornithodoros moubata OX=6938 GN=TSGP1 PE=2 SV=1	+	+	–	Secreted Lipocalin/Histamine binding
10	M9W8K9	TSGP4 OS=Ornithodoros moubata OX=6938 PE=2 SV=1	+	+	Amine binding	Secreted Lipocalin/Histamine binding
11	Q5YD59; B6E211	Complement inhibitor OS=Ornithodoros moubata OX=6938 GN=CI PE=1 SV=1;Putative complement inhibitor isoform 2 (Fragment) OS=Ornithodoros moubata OX=6938 PE=2	+	+	–	Secreted Lipocalin/C5 binding
12	Q6QD55; A0A1Z5L5D2	Cystatin-2 OS=Ornithodoros moubata OX=6938 PE=1 SV=1;Lysine-specific histone demethylase (Fragment) OS=Ornithodoros moubata OX=6938 PE=4 SV=1	+	+	Cysteine-type endopeptidase inhibitor activity	Secreted Protease inhibitor
13	Q6QZV3	Mucin/peritrophin-like protein OS=Ornithodoros moubata OX=6938 PE=2 SV=1	+	+	Chitin binding	Secreted Extracellular matrix
14	Q6QZV4	Putative thyropin OS=Ornithodoros moubata OX=6938 PE=2 SV=1	+	+	–	Secreted Protease inhibitor
15	Q8IT76	Alpha-2-macroglobulin splice variant 1 OS=Ornithodoros moubata OX=6938 PE=2 SV=1	+	+	Serine-type endopeptidase inhibitor activity	Secreted Protease inhibitor
16	A0A1Z5KZT7	Ran-binding protein RanBP (Fragment) OS=Ornithodoros moubata OX=6938 PE=3 SV=1	–	+	Deoxyribonuclease II activity	Housekeeping Nuclear regulation
17	A0A1Z5L6V3;A0A1Z5KYQ1	Uncharacterized protein (Fragment) OS=Ornithodoros moubata OX=6938 PE=3 SV=1	–	+	Cytokine activity	Secreted Cytokine activity
18	A0A1Z5L916	Beta-galactosidase (Fragment) OS=Ornithodoros moubata OX=6938 PE=3 SV=1	–	+	Beta-galactosidase activity	Housekeeping Metabolism/carbohydrates
19	A0A1Z5KY49	Salivary lipid interacting protein (Fragment) OS=Ornithodoros moubata OX=6938 PE=3 SV=1	–	+	–	Secreted Innate immunity Metabolism/lipids
20	A0A1Z5LBS2	Heme lipoprotein (Fragment) OS=Ornithodoros moubata OX=6938 PE=4 SV=1	–	+	Lipid transporter activity	Housekeeping Metabolism/heme-iron binding
21	A0A1Z5LH91	Histone H4 (Fragment) OS=Ornithodoros moubata OX=6938 PE=3 SV=1	–	+	Protein heterodimerization activity	Housekeeping Nuclear regulation
22	A0A1Z5LG77	Elongation factor 1-alpha (Fragment) OS=Ornithodoros moubata OX=6938 PE=3 SV=1	–	+	GTPase activity	Housekeeping Protein synthesis

Protein function was assigned with the UniProt’s Retrieve/ID mapping tool.

Underlined names indicate proteins that were identified in samples collected from both, *B. duttonii* infected and uninfected ticks.

**Names in bold font** indicate proteins identified only in samples collected from infected ticks.

## Discussion

The composition and number of proteins detected in sialomes of hematophagous arthropods are heavily influenced by multiple parameters, including such intrinsic factors as vector species, geographical origin of the studied population, sex, developmental stage, previous blood meal host(s), and inherent between-individual variability in expressed salivary proteins (Wang et al., 2001; Rohoušová et al., 2012; Díaz-Martín et al., 2013; Mans, 2016; Kim et al., 2016; Mans, 2019). Moreover, extraneous influences such as the choice of a technique used to stimulate salivation, storage and preparation of the collected samples, and reproducibility issues between different instruments all might contribute to the results of a proteomics study (Barker et al., 1973; Tabb et al., 2010; Díaz-Martín et al., 2013; Mans, 2019). This implies that all of the current approaches aimed at characterization of the incredibly complex and dynamic tick sialome likely will identify different subsets of proteins in the same type of samples, with none of them being comprehensive on their own but rather complementary to each other (Mans, 2020).

The total number of proteins detected in our study is significantly less than identified by earlier studies on the sialome of *O. moubata* (Díaz-Martín et al., 2013; Oleaga et al., 2021). These differences might be explained by the distinct approaches taken in each study. Thus, Díaz-Martín et al. (2013) identified 118 and 85 different proteins in pilocarpine-induced saliva samples collected from females and males, respectively, of which 69.5% and 40% became apparent only after the procedure known as protein equalization, aimed at diminishing content of the most abundant proteins in the analyzed samples. A follow-up study by the same research group utilizing the proteomics informed by transcriptomics (PIT) approach further increased the total number of salivary proteins identified in adult *O. moubata* of both sexes to 299 (Oleaga et al., 2021). However, a pertinent biological question remains, whether all of these diverse proteins identified up to date have some relevance at the tick-host interface? Some observations suggest that induced secretions obtained from extraneously stimulated ticks do not fully correspond to saliva secreted during the physiologically complex process of blood engorgement (Mans, 2019). In contrast, our approach represents unequivocal proof that the identified proteins are being secreted during the tick feeding.

Although the in-solution trypsin digestion used in our sample preparation protocol tend to detect fewer proteins than other methods, likely they are the most abundantly expressed ones (Kalume et al., 2005). This is further corroborated by the constant presence of lipocalins across all sample types analyzed in our study (**Supplementary Figure 1**), a family of proteins that had been previously shown to be hyperabundant in *O. moubata* saliva (Oleaga et al., 2021). On the other hand, it is unclear, whether the qualitative differences between the FUs can be solely attributed to the highly diluted nature of the analyte because our samples contained secretions of much smaller numbers of ticks than in the chemically stimulated *O. moubata* sialomes [secretions from 6-13 nymphs vs pooled saliva from ~68-97 adults, as per yields indicated by Díaz-Martín et al (Díaz-Martín et al., 2013)], or they hint to the individual variability in saliva composition, which can be obscured by pooling the secretions from multiple specimens (Rohoušová et al., 2012). So far, this phenomenon has been reported only in a few ixodid species (Wang et al., 2001; Kim et al., 2016; Nuttall, 2019) and was hypothesized to play a role in the ability of ticks to successfully feed on multiple types of hosts (Wang et al., 2001), which is consistent with the indiscriminate feeding habits of soft ticks. Another, non-mutually exclusive explanation is that the observed polymorphism in salivary proteins can act as a sort of “antigenic variation” in the vector-host arms race and provides a basis for the evolution of gregarious feeding (Wang et al., 2001), behavior which is also known to occur in argasids (Kim et al., 2017; De Oliveira et al., 2020). Interestingly, the altered transmission potential in some arbovirus-vector combinations depending on the number of simultaneously feeding ticks (Whitman and Aitken, 1960; Miller et al., 1985; Pereira de Oliveira et al., 2019) might lend some support to the idea of individual variability in salivary secretions, given the importance of saliva assisted transmission for this group of pathogens (Nuttall, 2019).

In general, proteins that can be broadly classified as molecules involved in combat against essential at the feeding site host defenses such as hemostasis and activation of innate immunity, were well represented in both sample types (6 out of 10 shared proteins; **Table 2**). We will not discuss them in further detail because several excellent reviews have been published on this topic in recent years [i.e. see (Mans, 2019; Nuttall, 2019)]. Similarly, the presence of some “housekeeping” proteins, in tick salivary secretions (e.g. actin) could be confirmed, suggesting they indeed are being secreted through a non-canonical pathway, to play some yet to be identified functions at the feeding site (Díaz-Martín et al., 2013; Ribeiro and Mans, 2020). However, what genuinely stands out, is the identification of 5 proteins occurring solely in the samples collected from *B. duttonii* infected ticks, suggesting their expression was altered by the infection. These can be classified as proteins involved in amino acid, carbohydrate, and lipid metabolism (arginine kinase, beta-N-acetylhexosaminidase, and phospholipase B-like, respectively), translational machinery (60S ribosomal protein L40, or RPL40), and antioxidant defense (glutathione peroxidase). This can be explained by increased metabolic costs of the infection and related cellular stress, or activated immune response in the vector. For example, the regulatory role of RPL40 in stress response has been described in model organisms such as *Drosophila* fruit flies, or arginine kinase has been shown to contribute to the resistance of *Bombyx mori* to nucleopolyhedrovirus (Kang et al., 2011; Espinosa et al., 2017). Hence, the presence of these peptides does not appear to be unique to the tick infection with *B. duttonii* but the exact functions of functionally similar proteins in the tick-pathogen interactions remain to be elucidated.

On the other hand, as has been suggested for the Lyme disease spirochetes, borrelia can selectively manipulate the vector’s metabolism to ensure its survival and persistence in tick tissues (Cotté et al., 2014; Kim et al., 2021). In a vector-competent *Ornithodoros* tick, RF *Borrelia* reside within the salivary gland environment for prolonged periods (likely, its entire lifespan), which in the long-lived soft ticks potentially could last decades; however, it remains unclear if the replication of spirochetes occurs within the infected salivary glands and what sorts of nutrients might be required for them to successfully colonize and persist within the organ (Schwan and Piesman, 2002; Schwan, 2021). It is known, for example, that lipids are utilized by RF *Borrelia* during the *in vitro* growth and they can procure essential fatty acids through phospholipase B mediated hydrolysis of lysolecithin (Cutler, 2002) and infection of ticks with other pathogens, such as the intracellular alphaproteobacterium *Anaplasma phagocytophilum*, has been shown to modulate the vector’s lipid metabolism and host-derived protein contents in tick tissues, including phospholipases (Villar et al., 2015; Villar et al., 2016). Another important secondary source of energy in nutrient-poor environments (which is likely the case of salivary glands) could be the arginine dihydrolase (ADH) pathway, which occurs in many anaerobic bacteria, including spirochetes of the genus *Treponema* (Blakemore and Canale-Parola, 1976) and microaerophilic eukaryotes such as *Giardia duodenalis* (Brown et al., 1998). Interestingly, genes encoding all key components of the ADH pathway are present in RF *Borrelia*, whereas Lyme spirochetes possess only a truncated version of the pathway (Lescot et al., 2008; Lin et al., 2017; Richards et al., 2022), suggesting this could reflect the well-known differences in colonization strategies of the vector between these two groups of spirochetes (Schwan and Piesman, 2002). Albeit, the exact mechanisms behind such intertwined metabolism between the pathogen and its vector must be scrutinized in future studies.

Finally, glutathione peroxidase is a homolog of SALP25D from *Ixodes scapularis*, which is a well-characterized antioxidant in the hard tick saliva that has been shown to protect *Borrelia burgdorferi* from killing by reactive oxygen species and facilitate its acquisition by the feeding vector (Narasimhan et al., 2007). Interestingly, a recent thorough study by Bourret et al. (2019) characterized salivary glands in another soft tick species, *Ornithodoros turicata*, which is a vector of the North American RF species *B. turricatae*, as a highly oxidative environment. Their transcriptomic analysis showed that genes responsible for antioxidant defenses, including *gpx* (encoding for glutathione peroxidase) were abundantly expressed although the authors could not detect any significant differences between salivary glands of borrelia infected vs uninfected ticks. Furthermore, it remained inconclusive whether the spirochetes could benefit from the presence of the antioxidant enzymes because they are expected to localize within the cellular environment of the salivary gland acini (Bourret et al., 2019). Our finding of glutathione peroxidase in naturally expectorated saliva from infected ticks confirms that the protein is secreted in the saliva of *O. moubata* and adds an interesting twist to the story, suggesting that in contrast to *B. turricatae*, the agent of African RF, *B. duttonii*, might partially rely on the antioxidant defense from its vector during the persistent colonization of the salivary glands.

We present a straightforward approach to collect naturally expectorated tick saliva and identify proteinaceous components present at the tick-host interface, which is an important addition to the toolbox available to study the vector-host-pathogen interactions in soft ticks. Although there are known interspecies differences in feeding behavior among argasids, it seems that glutathione elicits a uniform feeding response, which has been observed in at least 5 soft tick species., namely *O. tholozani*, *O. moubata*, *Argas persicus* (Galun and Kindler, 1965; Ben-Yakir and Galun, 1993), *O. coriaceus* (Hokama et al., 1987) and *O. turicata* (SF, unpublished data). Interestingly, the subsequent fate of the ticks is rarely reported but in our experiments, the majority of *O. moubata* that gorged on the GSH solution died within a few weeks, whereas in *O. coriaceus* (Hokama et al., 1987) and *O. turicata* the diet did not affect tick mortality. Our approach could also be used to study salivary secretions in other rapidly feeding hematophagous vectors (Sri-In et al., 2020) but currently, its application is lagging behind that in phytophagous arthropods, such as aphids and spider mites (Jonckheere et al., 2016; van Bel and Will, 2016). There is also room for improvement as the experimental setup might need to be adapted to sufficiently feed different life stages/species of ticks, or to increase the concentration of proteins in collected saliva samples. Nevertheless, our result is proof of concept that meaningful data pertaining to pathogen transmission by soft ticks can be collected in a more physiological manner than with currently accepted methods.

## Data availability statement

The data presented in the study are deposited in the ProteomeXchange repository, accession number PXD038824. The data can be found at Project Webpage: http://www.ebi.ac.uk/pride/archive/projects/PXD038824 and FTP Download at: ftp://ftp.pride.ebi.ac.uk/pride/data/archive/2023/01/PXD038824.

## Author contributions

SF, JS and ROMR conceived and designed the study. SF and ROMR conducted the experiments and wrote the manuscript. FD conducted the LC-MS analyses. All authors contributed to the article and approved the submitted version.

## Supplementary material

Supplementary Figure 1 A heatmap showing the relative intensities (LFQ values) of the detected proteins across the samples.
