# Supplementary figures and images for: A simple non-invasive method to collect soft tick saliva reveals differences in Ornithodoros moubata saliva composition between ticks infected and uninfected with Borrelia duttonii spirochetes
Source: Front Cell Infect Microbiol. 2023 Jan 20;13:1112952. doi: 10.3389/fcimb.2023.1112952 (PMC9895398; doi:10.3389/fcimb.2023.1112952)

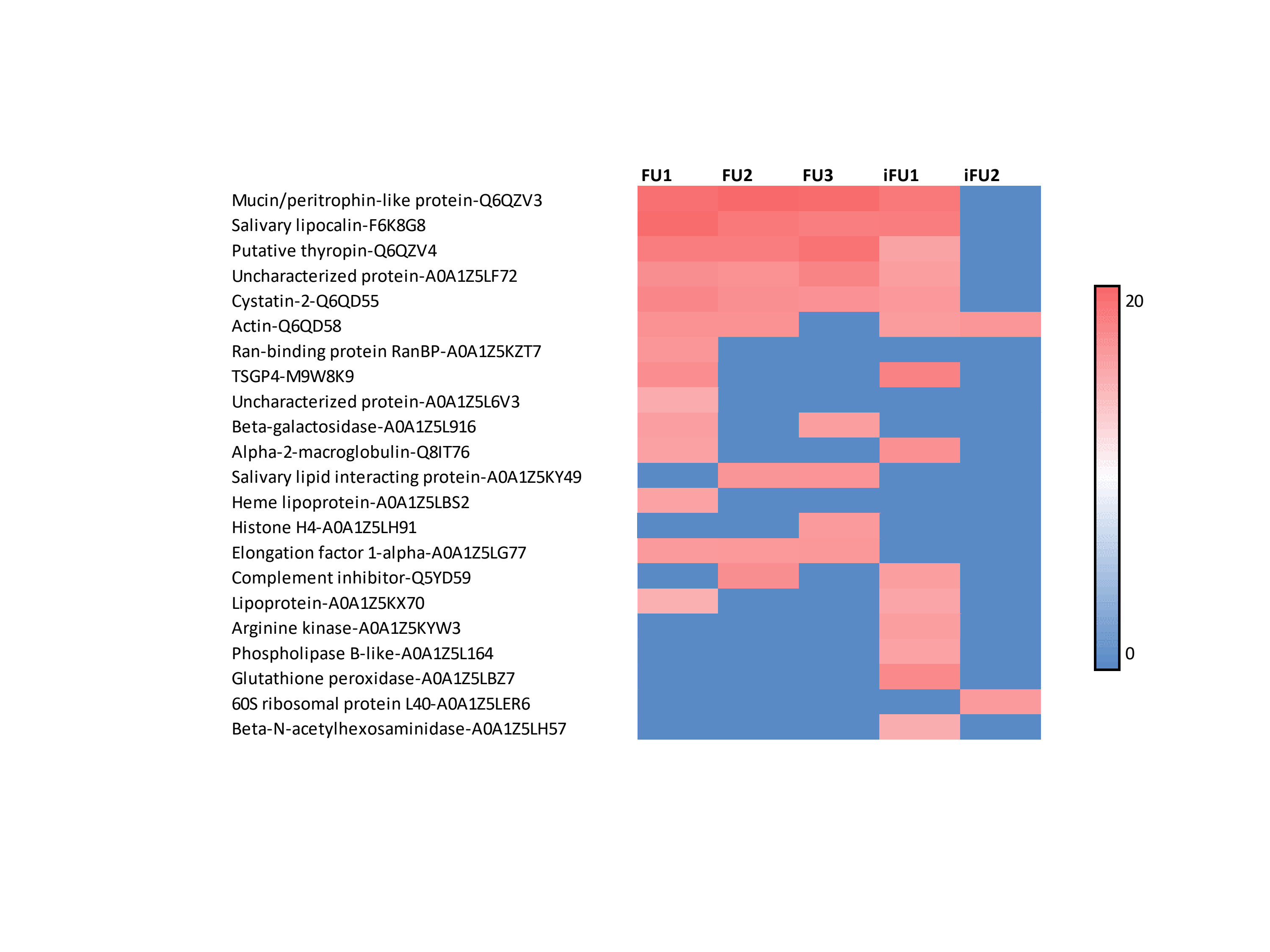

Supplement: Supplementary Figure 1 — A heatmap showing the relative intensities (LFQ values) of the detected proteins across the samples. [file Image_1.tif]
